# Supplementary material for: Subtle selectivity in a pheromone sensor triumvirate desynchronizes competence and predation in a human gut commensal
Source: eLife. 2019 Aug 21;8:e47139. doi: 10.7554/eLife.47139 (PMC6703854; doi:10.7554/eLife.47139)
Supplement: Supplementary file 1. [file elife-47139-supp1.docx]

## Supplementary File 1-5

## List of bacterial strains used in this study.

|  | Characteristics | | Reference/source |
| --- | --- | --- | --- |
| *Escherichia coli* | | |  |
| TOP10 | *mcrA*, Δ(*mrr-hsd*RMS-*mcrBC*), Phi80*lacZ(del)M15*, Δ*lacX74*, *deoR*, *recA1*, *araD139*, Δ(*ara-leu*)7697, *galU*, *galK*, *rpsL(SmR)*, *endA1*, *nupG* | | Invitrogen |
| *Streptococcus salivarius* | |  |  |
| HSISS4 | Wild-type gastro-intestinal tract isolate | | (Van den Bogert et al., 2014) |
| JM1004 | HSISS4 Δ*comA::cat* | | (Mignolet et al., 2018) |
| JM1013 | HSISS4 Δ*slv5* | | (Mignolet et al., 2018) |
| JM1019 | HSISS4 *tRNA^Thr^::*P*_comS_-luxAB*-*cat* | | (Mignolet et al., 2018) |
| JM1020 | HSISS4 *tRNA^Thr^::*P*_comX_-luxAB*-*cat* | | (Mignolet et al., 2018) |
| JM1021 | HSISS4 *tRNA^Thr^::*P*_blpK_-luxAB*-*cat* | | (Mignolet et al., 2018) |
| JM1022 | HSISS4 *tRNA^Thr^::*P*_HSISS4_00176_-luxAB*-*cat* | | (Mignolet et al., 2018) |
| JM1023 | HSISS4 *tRNA^Thr^::*P*_HSISS4_01584_-luxAB*-*cat* | | (Mignolet et al., 2018) |
| JM1024 | HSISS4 *tRNA^Thr^::*P*_slvV_-luxAB*-*cat* | | (Mignolet et al., 2018) |
| JM1025 | HSISS4 *tRNA^Thr^::*P*_blpG_-luxAB*-*cat* | | (Mignolet et al., 2018) |
| JM1026 | HSISS4 *tRNA^Thr^::*P*_slvW_-luxAB*-*cat* | | (Mignolet et al., 2018) |
| JM1027 | HSISS4 *tRNA^Thr^::*P*_slvX_-luxAB*-*cat* | | (Mignolet et al., 2018) |
| JM1028 | HSISS4 *tRNA^Thr^::*P*_slvY_-luxAB*-*cat* | | (Mignolet et al., 2018) |
| JM1100 | HSISS4 *tRNA^Thr^::*P*_sptA_-luxAB*-*cat* | | This work |
| JM1101 | HSISS4 *tRNA^Ser^::*P*_32_-scuR*-Spec^R^ (*scuR^++^*) | | This work |
| JM1102 | HSISS4 *tRNA^Ser^::*P*_32_-sarF-ST*-Spec^R^ (*sarF^++^*) | | This work |
| JM1103 | JM1019 *tRNA^Ser^::*P*_32_-scuR*- Spec^R^ | | This work |
| JM1104 | JM1020 *tRNA^Ser^::*P*_32_-scuR*- Spec^R^ | | This work |
| JM1105 | JM1021 *tRNA^Ser^::*P*_32_-scuR*- Spec^R^ | | This work |
| JM1106 | JM1022 *tRNA^Ser^::*P*_32_-scuR*- Spec^R^ | | This work |
| JM1107 | JM1023 *tRNA^Ser^::*P*_32_-scuR*- Spec^R^ | | This work |
| JM1108 | JM1024 *tRNA^Ser^::*P*_32_-scuR*- Spec^R^ | | This work |
| JM1109 | JM1025 *tRNA^Ser^::*P*_32_-scuR*- Spec^R^ | | This work |
| JM1110 | JM1026 *tRNA^Ser^::*P*_32_-scuR*- Spec^R^ | | This work |
| JM1111 | JM1027 *tRNA^Ser^::*P*_32_-scuR*- Spec^R^ | | This work |
| JM1112 | JM1028 *tRNA^Ser^::*P*_32_-scuR*- Spec^R^ | | This work |
| JM1113 | JM1100 *tRNA^Ser^::*P*_32_-scuR*- Spec^R^ | | This work |
| JM1114 | JM1019 *tRNA^Ser^::*P*_32_-sarF*- Spec^R^ | | This work |
| JM1115 | JM1027 *tRNA^Ser^::*P*_32_-sarF*- Spec^R^ | | This work |
| JM1116 | JM1100 *tRNA^Ser^::*P*_32_-sarF*- Spec^R^ | | This work |
| JM1117 | JM1113 Δ*comR::ery* | | This work |
| JM1118 | HSISS4 Δ*scuR-sarF::ery* | | This work |
| JM1119 | JM1113 Δ*scuR-sarF::ery* | | This work |
| JM1120 | JM1116 Δ*scuR-sarF::ery* | | This work |
| JM1121 | HSISS4 *tRNA^Thr^::*P*_sptA_-cat*-Spec^R^ | | This work |
| JM1122 | HSISS4 *tRNA^Thr^::*P*_sptA_-cat*-*lox72* | | This work |
| JM1123 | JM1122 *tRNA^Ser^::*P*_xyl1_-comR*-Spec^R^ | | This work |
| JM1124 | JM1013 *tRNA^Thr^::*P*_sptA_-cat*-*lox72* | | This work |
| JM1125 | JM1122 *tRNA^Ser^::*P*_xyl2_*-*BH6*-Spec^R^ | | This work |
| JM1126 | JM1122 *tRNA^Ser^::*P*_xyl1_*-*BI1*- Spec^R^ | | This work |
| JM1127 | JM1122 *tRNA^Ser^::*P*_xyl1_*-*BI2*- Spec^R^ | | This work |
| JM1128 | JM1122 *tRNA^Ser^::*P*_xyl1_*-*BI5*- Spec^R^ | | This work |
| JM1129 | JM1122 *tRNA^Ser^::*P*_xyl1_*-*BI6*- Spec^R^ | | This work |
| JM1130 | JM1122 *tRNA^Ser^::*P*_xyl1_*-*BI7*- Spec^R^ | | This work |
| JM1131 | JM1122 *tRNA^Ser^::*P*_xyl1_*-*BI9*- Spec^R^ | | This work |
| JM1132 | JM1122 *tRNA^Ser^::*P*_xyl1_*-*BI10*- Spec^R^ | | This work |
| JM1133 | JM1122 *tRNA^Ser^::*P*_xyl1_*-*BI11*- Spec^R^ | | This work |
| JM1134 | JM1122 *tRNA^Ser^::*P*_xyl1_*-*BI12*- Spec^R^ | | This work |
| JM1135 | JM1122 *tRNA^Ser^::*P*_xyl2_*-*BJ1*- Spec^R^ | | This work |
| JM1136 | JM1122 *tRNA^Ser^::*P*_xyl1_*-*BK1*- Spec^R^ | | This work |
| JM1137 | JM1122 *tRNA^Ser^::*P*_xyl1_*-*BK2*- Spec^R^ | | This work |
| JM1138 | JM1122 *tRNA^Ser^::*P*_xyl1_*-*BK3*- Spec^R^ | | This work |
| JM1139 | JM1122 *tRNA^Ser^::*P*_xyl1_*-*BK4*- Spec^R^ | | This work |
| JM1140 | JM1122 *tRNA^Ser^::*P*_xyl1_*-*BK5*- Spec^R^ | | This work |
| JM1141 | JM1122 *tRNA^Ser^::*P*_xyl1_*-*BK6*- Spec^R^ | | This work |
| JM1142 | JM1122 *tRNA^Ser^::*P*_xyl1_*-*BK7*- Spec^R^ | | This work |
| JM1143 | JM1122 *tRNA^Ser^::*P*_xyl1_*-*BK8*- Spec^R^ | | This work |
| JM1144 | JM1122 *tRNA^Ser^::*P*_xyl1_*-*BK9*- Spec^R^ | | This work |
| JM1145 | JM1124 *tRNA^Ser^::*P*_xyl1_*-*BL1*- Spec^R^ | | This work |
| JM1146 | JM1124 *tRNA^Ser^::*P*_xyl1_*-*BL2*- Spec^R^ | | This work |
| JM1147 | JM1124 *tRNA^Ser^::*P*_xyl1_*-*BL3*- Spec^R^ | | This work |
| JM1148 | JM1124 *tRNA^Ser^::*P*_xyl2_*-*BM1*- Spec^R^ | | This work |
| JM1149 | JM1124 *tRNA^Ser^::*P*_xyl1_*-*BN1*- Spec^R^ | | This work |
| JM1150 | JM1124 *tRNA^Ser^::*P*_xyl1_*-*BN2*- Spec^R^ | | This work |
| JM1151 | JM1124 *tRNA^Ser^::*P*_xyl1_*-*BN3*- Spec^R^ | | This work |
| JM1152 | JM1124 *tRNA^Ser^::*P*_xyl1_*-*BN4*- Spec^R^ | | This work |
| JM1153 | JM1122 *tRNA^Ser^::*P*_xyl2_*-*BO1*- Spec^R^ | | This work |
| JM1154 | JM1122 *tRNA^Ser^::*P*_xyl2_*-*BO2*- Spec^R^ | | This work |
| JM1155 | JM1122 *tRNA^Ser^::*P*_xyl1_*-*BP1*- Spec^R^ | | This work |
| JM1156 | JM1100 *tRNA^Ser^::*P*_xyl1_*-*BI5*- Spec^R^ | | This work |
| JM1157 | JM1100 *tRNA^Ser^::*P*_xyl1_*-*BI7*- Spec^R^ | | This work |
| JM1158 | JM1100 *tRNA^Ser^::*P*_xyl1_*-*BI10*- Spec^R^ | | This work |
| JM1159 | JM1100 *tRNA^Ser^::*P*_xyl2_*-*BJ1*- Spec^R^ | | This work |
| JM1160 | JM1100 *tRNA^Ser^::*P*_xyl1_*-*BK1*- Spec^R^ | | This work |
| JM1161 | JM1100 *tRNA^Ser^::*P*_xyl1_*-*BK4*- Spec^R^ | | This work |
| JM1162 | JM1100 *tRNA^Ser^::*P*_xyl1_*-*BK8*- Spec^R^ | | This work |
| JM1163 | JM1100 *tRNA^Ser^::*P*_xyl1_*-*BK9*- Spec^R^ | | This work |
| JM1164 | JM1100 *tRNA^Ser^::*P*_xyl1_*-*BL2*- Spec^R^ | | This work |
| JM1165 | JM1100 *tRNA^Ser^::*P*_xyl1_*-*BL3*- Spec^R^ | | This work |
| JM1166 | JM1100 *tRNA^Ser^::*P*_xyl2_*-*BM1*- Spec^R^ | | This work |
| JM1167 | JM1100 *tRNA^Ser^::*P*_xyl1_*-*BN2*- Spec^R^ | | This work |
| JM1168 | JM1100 *tRNA^Ser^::*P*_xyl1_*-*BN3*- Spec^R^ | | This work |
| JM1169 | JM1100 *tRNA^Ser^::*P*_xyl2_*-*BO2*- Spec^R^ | | This work |
| JM1170 | JM1100 *tRNA^Ser^::*P*_xyl1_*-*BP1*- Spec^R^ | | This work |
| JM1171 | JM1157 Δ*scuR::ery* | | This work |
| JM1172 | JM1157 Δ*sarF::ery* | | This work |
| JM1173 | JM1157 Δ*scuR-sarF::ery* | | This work |
| JM1174 | JM1157 Δ*comR::ery* | | This work |
| JM1175 | JM1100 Δ*scuR::ery* | | This work |
| JM1176 | JM1100 Δ*sarF::ery* | | This work |
| JM1177 | JM1027 Δ*scuR::ery* | | This work |
| JM1178 | JM1027 Δ*sarF::ery* | | This work |
| JM1179 | JM1019 Δ*scuR::ery* | | This work |
| JM1180 | JM1019 Δ*sarF::ery* | | This work |
| JM1181 | HSISS4 *tRNA^Thr^::*P*_sptA_^CT🡪AC^-luxAB*-*cat* | | This work |
| JM1182 | HSISS4 *tRNA^Thr^::*P*_sptA_^+1^-luxAB*-*cat* | | This work |
| JM1183 | HSISS4 *tRNA^Thr^::*P*_sptA_^+A^-luxAB*-*cat* | | This work |
| JM1184 | HSISS4 *tRNA^Thr^::*P*_comX_^G🡪A^-luxAB*-*cat* | | This work |
| JM1185 | JM1101 Δ*comA::cat* | | This work |
| JM1186 | JM1101 Δ*sptA::cat* | | This work |
| JM1187 | HSISS4 Δ*scuR* | | This work |
| JM1188 | HSISS4 Δ*sarF* | | This work |
| JM1189 | HSISS4 Δ*sptA::cat* | | This work |
| JM1190 | JM1019 Δ*scuR-sarF::ery* | | This work |
| JM1191 | JM1021 Δ*scuR-sarF::ery* | | This work |
| JM1192 | JM1027 Δ*scuR-sarF::ery* | | This work |
| JM1193 | JM1100 Δ*scuR-sarF::ery* | | This work |
| JM1194 | HSISS4 *tRNA^Thr^::*P*_ssbA_-luxAB*-*cat* | | This work |
| JM1195 | HSISS4 *tRNA^Thr^::*P*_dprA_-luxAB*-*cat* | | This work |
| JM1196 | HSISS4 *tRNA^Thr^::*P*_comEA_-luxAB*-*cat* | | This work |
| JM1197 | HSISS4 *tRNA^Thr^::*P*_comFA_-luxAB*-*cat* | | This work |
| JM1198 | HSISS4 *tRNA^Thr^::*P*_cbpD1_-luxAB*-*cat* | | This work |
| JM1199 | JM1020 Δ*scuR::ery* | | This work |
| JM1200 | JM1020 Δ*sarF::ery* | | This work |
| JM1201 | JM1020 Δ*scuR-sarF::ery* | | This work |
| JM1202 | JM1021 Δ*scuR::ery* | | This work |
| JM1203 | JM1021 Δ*sarF::ery* | | This work |
| JM1204 | JM1021 Δ*scuR-sarF::ery* | | This work |
| JM1205 | JM1024 Δ*scuR::ery* | | This work |
| JM1206 | JM1024 Δ*sarF::ery* | | This work |
| JM1207 | JM1024 Δ*scuR-sarF::ery* | | This work |
| JM1208 | JM1026 Δ*scuR::ery* | | This work |
| JM1209 | JM1026 Δ*sarF::ery* | | This work |
| JM1210 | JM1026 Δ*scuR-sarF::ery* | | This work |
| JM1211 | JM1027 Δ*scuR::ery* | | This work |
| JM1212 | JM1027 Δ*sarF::ery* | | This work |
| JM1213 | JM1027 Δ*scuR-sarF::ery* | | This work |
| JM1214 | JM1028 Δ*scuR::ery* | | This work |
| JM1215 | JM1028 Δ*sarF::ery* | | This work |
| JM1216 | JM1028 Δ*scuR-sarF::ery* | | This work |
| *Lactococcus lactis* subsp. *lactis* | | |  |
| IL1403 | Laboratory strain | | (Chopin et al., 1984) |

## List of plasmids used in this study.

|  | Characteristics | Reference/source |
| --- | --- | --- |
| pGhostcre | Thermosensitive replication vector in *S. salivarius*, encoding the Cre recombinase; ery^R^ | (Fontaine et al., 2010) |
| pGIUD0855*ery* | pUC18 derivative containing the *erm* gene | (Fontaine et al., 2010) |
| pSEUDO-P*_usp45_*-*_sf_gfp*(Bs) | *erm-oroP* containing vector | (Overkamp et al., 2013) |
| pGILF*spec* | pG^+^host9 derivative containing the spectinomycin resistance cassette P_Spec_^R^*-*Spec^R^ downstream of *luxAB* | (Haustenne et al., 2015) |
| pJUD*specmut1* | pGILF*spec* derivative in which a *Spe*I restriction site was mutated | (Mignolet et al., 2018) |
| pJUD*specmut1-gfp^+^*ter | Terminator associated*-gfp^+^* ORF cloned in pJUD*specmut1* | (Mignolet et al., 2018) |
| pNZ5319 | pACYC184 derivative containing the *cat* gene under the control of the P32 constitutive promoter from *Lactococcus lactis* | (Lambert et al., 2007) |
| pJIM*cat* | pG^+^host9 containing the *luxAB* genes of *Photorhabdus luminescens,* and a *cat* cassette | (Mignolet et al., 2018) |
| pBAD-*comR-ST* | pBAD*hisA* derivative encoding ComR fused to a C-terminal StreptagII | (Mignolet et al., 2018) |
| pBAD-*scuR-ST* | pBAD*hisA* derivative encoding ScuR fused to a C-terminal StreptagII | This work |
| pBAD-*sarF-ST* | pBAD*hisA* derivative encoding SarF fused to a C-terminal StreptagII | This work |
|  |  |  |

## List of oligonucleotides used in this study.

| Names | Sequences |
| --- | --- |
| rggD_NcoI SS | 5’-AAAAAACCATGGCAGAAGATATTAAAATCAAGA-3’ |
| rggD_MunI | 5’-AAAAAACAATTGCTTTGACTCGTTACTTGTAT-3’ |
| rggC_NcoI SS | 5’-AAAAAACCATGGCTGAAGATATTAAAATCGAGA-3’ |
| rggC_RI SS | 5’-AAAAAAGAATTCTTCTATATTTAAATCTTTTT-3’ |
| Uplox66 | 5’-TAAGGAAGATAAATCCCATAAGG-3’ |
| DNlox71 | 5’-TTCACGTTACTAAAGGGAATGTA-3’ |
| lox66-ery | 5’-TAAGGAAGATAAATCCCATAA  GGTACCTAATAATTTATCTACATTCC-3’ |
| lox71-ery | 5’-TTCACGTTACTAAAGGGAA  TGTAAAATGATACACCAATCAGTGC-3’ |
| F_spec | 5’-TAATAAGGCCGGCCAATAAA-3’ |
| R_spec | 5’-ATAGGATGAGAACTCCCATG-3’ |
| UPery-oroP | 5’-AAGGTTGATGTTACTGCTGATA-3’ |
| DNery-oroP | 5’-TGCTGACTTGCACCATATCATA-3’ |
| UF_tRNAser | 5’-CAAGATTAACCATGACCTTC-3’ |
| UR_tRNAser | 5’-AGTAATTAAAAAGAAGATGG-3’ |
| DF_tRNAser | 5’-TACCTAAAAAGTGTCCCTTC-3’ |
| DR2_tRNAser | 5’-TTGGATAAGGTCTTGACTTC-3’ |
| UF_tRNAthr | 5’-TGTCAAAGGATTAGGAAAAC-3’ |
| UR_tRNAthr | 5’-TTGATTTATACCTCTCAATTT-3’ |
| DF_tRNAthr | 5’-AAATCAACCTCTTTGAACATA-3’ |
| DR_tRNAthr | 5’-AAAAAAGAATTCATTCATGATGAGCGGGTTCGTGAGA-3’ |
| F_pZX9 | 5’-CCATCTTCTTTTTAATTACTTCTAGATTATATATGATATGATC-3’ |
| R_pZX9_ATG | 5’-CATATTTACCTCCTTTGATTTA-3’ |
| F_luxAB_ATG | 5’-ATGAAATTTGGAAACTTTTTGC-3’ |
| R_cat_tRNAthr | 5’-TATGTTCAAAGAGGTTGATTTCACGTTACTAAAGGGAATGTA-3’ |
| F_pept_xyl | 5’-TAAATCAAAGGAGGTAAATATGATCGCAATCCTANNNNNN  NNNNNNNNNNNNNNNTGATAATAAGGCCGGCCAATAAA-3’ |
| UFcomRJIM-SS1-4 | 5’-GCAGTACCACTCTATGCTAAATTTGCCAACTTTGA-3’ |
| URcomRJIM-SS1-4 | 5’-CCTTATGGGATTTATCTTCCTTAGAGACACTCCTTTATTTC-3’ |
| DFcomRJIM-SS1-4 | 5’-TACATTCCCTTTAGTAACGTGAAAAATGGTGGTGACATAAA-3’ |
| DRcomRJIM-SS1-4 | 5’-TGACGTGATTTCACCAGTACGACGTGAACTAAAGA-3’ |
| Up_comR SS1-4 | 5’-TTGCTTACAGTTGCTATGGT-3’ |
| Down_comR_SS1-4 | 5’-TCATCACAATGGTCACATCT-3’ |
| UFrggC JIM | 5’-AAAACTGCAAGTAGAGTCGCCGAATTAGAA-3’ |
| URrggC_JIM | 5’-CCTTATGGGATTTATCTTCCTTAACATAATTCCTTATGATTTAGA-3’ |
| DFrggC JIM | 5’-TACATTCCCTTTAGTAACGTGAAGACATTGATGTCCTTTTGA-3’ |
| DRrggD SS | 5’-TAGCTTCATTCATGTCATGTGTCGTCAAAA-3’ |
| Up_rggC JIM | 5’-AAATATCGTCATTGCCAGTA-3’ |
| Down_rggD SS | 5’-CTGAATAAGTTCAGCAGGTT-3’ |
| Down_rggD SS | 5’-TCACTTTGACTCGTTACTTGTGATTTTAATATCTTCTGACAT-3’ |
| rggD_S4_3 | 5’-ATGTCAGAAGATATTAAAATCACAAGTAACGAGTCAAAGTGA-3’ |
| rggD_S4_4 | 5’-TATCAGCAGTAACATCAACCTTCATGTCATGTGTCGTCAAAA-3’ |
| rggD_S4_5 | 5’-TATGATATGGTGCAAGTCAGCATCATGAAGTCTCCTGTCTAT-3’ |
| rggD_S4_6 | 5’-GGCTAGTACAGTAGCTGTAT-3’ |
| UFrggC SS | 5’-CTGTTTAGCCCTATCTTTGAGTTTATCAGT-3’ |
| URrggD JIM | 5’-CCTTATGGGATTTATCTTCCTTATGTATTCCCCTTGAGTTTT-3’ |
| DFrggD JIM | 5’-TACATTCCCTTTAGTAACGTGAAGAAAATATATCAGCAACAT-3’ |
| DRrggC SS | 5’-CAGTGGTTTGACGTTGTTTTTGAATACGGT-3’ |
| Up_rggC SS | 5’-AAGGTAGCCTAAACAACTCA-3’ |
| Down_rggC SS | 5’-TTTATTGGTACCAAACGCCA-3’ |
| rggC_S4_2 | 5’-CTATTCTATATTTAAATCTTTGATTTTAATATCTTCAGACAT-3’ |
| rggC_S4_3 | 5’-ATGTCTGAAGATATTAAAATCAAAGATTTAAATATAGAATAG-3’ |
| rggC_S4_4 | 5’-TATCAGCAGTAACATCAACCTTGACGTTGTTTTTGAATACGGT-3’ |
| rggC_S4_5 | 5’-TATGATATGGTGCAAGTCAGCAACTAGACATTCCTGAAGACT-3’ |
| rggC_S4_6 | 5’-TCCGCTAGTAGGATAGCTTT-3’ |
| UF_PcomR_luxAB | 5’-TAATTGAGGAGGTCTATGAG-3’ |
| UR_comA | 5’-CCTTATGGGATTTATCTTCCTTAATATGGATATTTTGACATGG-3’ |
| DF_comA | 5’-TACATTCCCTTTAGTAACGTGAAGCTAATTTCAATCCATTCCAG-3’ |
| DR_comA | 5’-ACAGTACTCTTTATTTGGTG-3’ |
| F_comR | 5’-CTAGAGGAGGAATTTAGATGAACATAAAAGACAGCATTG -3’ |
| Down_PcomS_JIMSS1-4 | 5’-GACAAAGTAGTCAAGACCGT-3’ |
| UF_potA2 | 5’-ATACTATACCTTTCAATGTC-3’ |
| UR_potA2 | 5’-CCTTATGGGATTTATCTTCCTTAATAAGGTTTGTCATATCTTG-3’ |
| DF_potA2 | 5’-TACATTCCCTTTAGTAACGTGAAGGAAAACTTAATGTTTAACC-3’ |
| DR_potA2 | 5’-ACTGATCCCTGAAAGCATTG-3’ |
| Up_potA2 | 5’-AGAGTATACCTTAAATGACC-3’ |
| Down_potA2 | 5’-GATTTAAAGATTTCGTGAAC-3’ |
| F_PpotA2_tRNAthr | 5’-AAATTGAGAGGTATAAATCAATCATTTTGGAAGCAAAATAC-3’ |
| R_PpotA2_luxAB_ATG | 5’-GCAAAAAGTTTCCAAATTTCATATCTTGATTTCTCCAATTTG-3’ |
| F_rggD | 5’-CTAGAGGAGGAATTTAGATGTCAGAAGATATTAAAATC-3’ |
| R_rggD | 5’-TTTATTGGCCGGCCTTATTATCACTTTGACTCGTTACTTG-3’ |
| F_rggC | 5’-CTAGAGGAGGAATTTAGATGTCTGAAGATATTAAAATC-3’ |
| R_StrepTag | 5’-TTTATTGGCCGGCCTTATTACTATTTCTCGAACTGCGG-3’ |
| F_P32-gfp+ter_spec | 5’- CCATCTTCTTTTTAATTACTGTCCTCGGGATATGATAAG -3’ |
| R_P32 | 5’-CATCTAAATTCCTCCTCTAG-3’ |
| F_cat_ATG | 5’-ATGAACTTTAATAAAATTGATT-3’ |
| R_cat_(spec) | 5’-TTTATTGGCCGGCCTTATTATAAAAGCCAGTCATTAGGC-3’ |
| R_PpotA2_cat_ATG | 5’-AATCAATTTTATTAAAGTTCATATCTTGATTTCTCCAATTTG-3’ |
| Pxyl_seq | 5’-TTGTTTATCCTCCTCTAGTC-3’ |
| spec2 | 5’-AACTCCTGATCCAAACATGTA-3’ |
| Cy3_F_PpotA2_EMSA | 5’-Cy3-TAACGAGTCAAAGTGACATAGATGTCCTTTTGATTCGTTA-3’ |
| R_PpotA2_EMSA | 5’-TAACGAATCAAAAGGACATCTATGTCACTTTGACTCGTTA-3’ |
| Cy3_F_P01665_EMSA | 5’-Cy3-CTCCATAGTGACATTTATGTCACTATTTTT-3’ |
| R_P01665_EMSA | 5’-AAAAATAGTGACATAAATGTCACTATGGAG-3’ |
| Cy3_F_PcomS_EMSA | 5’-Cy3-AATGGTGGTGACATAAATGTCACTACTTTT-3’ |
| R_PcomS_EMSA | 5’-AAAAGTAGTGACATTTATGTCACCACCATT-3’ |
| Cy3_F_PcomX_EMSA | 5’-Cy3-TTTTATAGTGACATATATGTCGCTATTTTA-3’ |
| R_PcomX_EMSA | 5’-TAAAATAGCGACATATATGTCACTATAAAA-3’ |
| Cy3_F_PcomX_EMSArev | 5’-Cy3-TTTTATAGTGACATATATGTCACTATTTTA-3’ |
| R_PcomX_EMSArev | 5’-TAAAATAGTGACATATATGTCACTATAAAA-3’ |
| F_PcomX_mut | 5’-CATATATGTCACTATTTTATT-3’ |
| R_PcomX_mut | 5’-AATAAAATAGTGACATATATG-3’ |
| F_PpotA2_mut2 | 5’-ACATAGATGTCACTTTGATTCGT-3’ |
| R_PpotA2_mut2 | 5’-ACGAATCAAAGTGACATCTATGT-3’ |
| F_PpotA2_mut+1 | 5’-TGATTCGTTATTTTTTTTGTTT-3’ |
| R_PpotA2_mut+1 | 5’-AAACAAAAAAAATAACGAATCA-3’ |
| F_PpotA2_mut+A | 5’-CATAGATGTCACTTTTGATTC-3’ |
| R_PpotA2_mut+A | 5’-GAATCAAAAGTGACATCTATG-3’ |
| F_PssbA_tRNAthr | 5’-AAATTGAGAGGTATAAATCAATTCATGGGAAATTGTGGTTC-3’ |
| R_PssbA_luxAB_ATG | 5’-GCAAAAAGTTTCCAAATTTCATTTTTTTACCTCCACC-3’ |
| F_PdprA_tRNAthr | 5’-AAATTGAGAGGTATAAATCAATGTTCTTGTGCTAAATTTGC-3’ |
| R_PdprA_luxAB_ATG | 5’-GCAAAAAGTTTCCAAATTTCATATAGATACCTCCTTTTC-3’ |
| F_PcomEA_tRNAthr | 5’-GCAAAAAGTTTCCAAATTTCACGCTATTCTCCTTAATTG-3’ |
| R_PcomEA_luxAB_GTG | 5’-GCAAAAAGTTTCCAAATTTCACGCTATTCTCCTTAATTG-3’ |
| F_PcomFA_tRNAthr | 5’-GCAAAAAGTTTCCAAATTTCATATCTTTCTATTCGAAAATA-3’ |
| R_PcomFA_luxAB_ATG | 5’-GCAAAAAGTTTCCAAATTTCATATCTTTCTATTCGAAAATA-3’ |
| F_PcbpD1_tRNAthr | 5’-GCAAAAAGTTTCCAAATTTCATTTTAACCTCCACCTAC-3’ |
| R_PcbpD1_luxAB_ATG | 5’-GCAAAAAGTTTCCAAATTTCATTTTAACCTCCACCTAC-3’ |

**List of EMSA annealed primers, overlapping and cloning PCR subfragments amplified in this study.**

| PCR/annealing | Primer 1 | Primer 2 |
| --- | --- | --- |
| *scuR* amplification for pBAD-*scuR-ST* cloning | rggD_NcoI SS | rggD_MunI |
| *sarF* amplification for pBAD-*sarF-ST* cloning | rggC_NcoI SS | rggC_RI SS |
| P*_32_-cat* cassette amplification | Uplox66 | DNlox71 |
| *Erm* cassette amplification | lox66-ery | lox71-ery |
| Spec^R^ cassette amplification for *tRNA^Ser^* locus | F_spec | R_spec |
| *erm-oroP* cassette amplification | UPery-oroP | DNery-oroP |
| P*_xyl1_* amplification | F_pZX9 | R_pZX9_ATG |
| P*_xyl2_* amplification | F_pZX9 | R_pZX9_ATG |
| *luxAB*-*cat* amplification | F_luxAB_ATG | R_cat_tRNAthr |
| P*_32_* amplification | F_P32-gfp+ter_spec | R_P32 |
| Spec^R^ cassette amplification for *tRNA^Thr^* locus | F_spec | R_cat_tRNAthr |
| Upstream homologous region of *tRNA^Ser^* locus | UF_tRNAser | UR_tRNAser |
| Downstream homologous region of *tRNA^Ser^* locus | DF_tRNAser | DR2_tRNAser |
| Upstream homologous region of *tRNA^Thr^* locus | UF_tRNAthr | UR_tRNAthr |
| Downstream homologous region of *tRNA^Thr^* locus | DF_tRNAthr | DR_tRNAthr |
| *scuR* amplification for P*_32_-scuR* fusion at *tRNA^Ser^* locus | F_rggD | R_rggD |
| *sarF-ST* amplification for P*_32_-sarF-ST* fusion at *tRNA^Ser^* locus | F_rggC | R_StrepTag |
| Promoter of *sptA* for *luxAB* fusion | F_PpotA2_tRNAthr | R_PpotA2_luxAB_ATG |
| Promoter of *sptA^CT🡪AC^* for *luxAB* fusion | F_PpotA2_mut2 | R_PpotA2_mut2 |
| Promoter of *sptA^+1^* for *luxAB* fusion | F_PpotA2_mut+1 | R_PpotA2_mut+1 |
| Promoter of *sptA^+A^* for *luxAB* fusion | F_PpotA2_mut+A | R_PpotA2_mut+A |
| Promoter of *comX^G🡪A^* for *luxAB* fusion | F_PcomX_mut | R_PcomX_mut |
| Promoter of *sptA* for *cat* fusion (screen) | F_PpotA2_tRNAthr | R_PpotA2_cat_ATG |
| *cat* cassette amplification for P*_sptA_* fusion (screen) | F_cat_ATG | R_cat_(spec) |
| Random peptide gene and Spec^R^ for *tRNA^Ser^* locus | F_pept_xyl | R_spec |
| Diagnostic PCR for random peptide sequencing | Pxyl_seq | spec2 |
| PCR for random peptide backcross | UF_tRNAser | DR2_tRNAser |
| PCR for *tRNA^Ser^::*P*_xyl1_-comR*- Spec^R^ amplification | UF_tRNAser | DR2_tRNAser |
| Upstream homologous region of *scuR* gene | UFrggC JIM | URrggC_JIM |
| Downstream homologous region of *scuR* gene | DFrggC JIM | DRrggD SS |
| Upstream homologous region of *scuR* gene (in-frame deletion) | UFrggC JIM | rggD_S4_2 |
| Downstream homologous region1 of *scuR* gene (in-frame deletion) | rggD_S4_3 | rggD_S4_4 |
| Downstream homologous region2 of *scuR* gene (in-frame deletion) | rggD_S4_5 | rggD_S4_6 |
| Diagnostic PCR for *scuR* deletion | Up_rggC JIM | Down_rggD SS |
| Upstream homologous region of *sarF* gene | UFrggC SS | URrggD JIM |
| Downstream homologous region of *sarF* gene | DFrggD JIM | DRrggC SS |
| Upstream homologous region of *sarF* gene (in-frame deletion) | UFrggC SS | rggC_S4_2 |
| Downstream homologous region1 of *sarF* gene (in-frame deletion) | rggC_S4_3 | rggC_S4_4 |
| Downstream homologous region2 of *sarF* gene (in-frame deletion) | rggC_S4_5 | rggC_S4_6 |
| Diagnostic PCR for *sarF* deletion | Up_rggC SS | Down_rggC SS |
| Diagnostic PCR for *scuR-sarF* deletion | Up_rggC JIM | Down_rggC SS |
| Upstream homologous region of *comR* gene | UFcomRJIM-SS1-4 | URcomRJIM-SS1-4 |
| Downstream homologous region of *comR* gene | DFcomRJIM-SS1-4 | DRcomRJIM-SS1-4 |
| Diagnostic PCR for *comR* deletion | Up_comR SS1-4 | Down_comR_SS1-4 |
| Upstream homologous region of *comA* gene | UF_PcomR_luxAB | UR_comA |
| Downstream homologous region of *comA* gene | DF_comA | DR_comA |
| Diagnostic PCR for *comA* deletion | F_comR | Down_PcomS_JIMSS1-4 |
| Upstream homologous region of *sptA* gene | UF_potA2 | UR_potA2 |
| Downstream homologous region of *sptA* gene | DF_potA2 | DR_potA2 |
| Diagnostic PCR for *sptA* deletion | Up_potA2 | Down_potA2 |
| Promoter of *sptA* annealing for EMSA | Cy3_F_PpotA2_EMSA | R_PpotA2_EMSA |
| Promoter of *slvX* annealing for EMSA | Cy3_F_P01665_EMSA | R_P01665_EMSA |
| Promoter of *comS* annealing for EMSA | Cy3_F_PcomS_EMSA | R_PcomS_EMSA |
| Promoter of comX annealing for EMSA | Cy3_F_PcomX_EMSA | R_PcomX_EMSA |
| Promoter of *comX^G🡪A^* annealing for EMSA | Cy3_F_PcomX_EMSArev | R_PcomX_EMSArev |
| Promoter of *ssbA* for *luxAB* fusion | F_PssbA_tRNAthr | R_PssbA_luxAB_ATG |
| Promoter of *dprA* for *luxAB* fusion | F_PdprA_tRNAthr | R_PdprA_luxAB_ATG |
| Promoter of *comEA* for *luxAB* fusion | F_PcomEA_tRNAthr | R_PcomEA_luxAB_GTG |
| Promoter of *comFA* for *luxAB* fusion | F_PcomFA_tRNAthr | R_PcomFA_luxAB_ATG |
| Promoter of *cbpD1* for *luxAB* fusion | F_PcbpD1_tRNAthr | R_PcbpD1_luxAB_ATG |

*Plasmid and Linear DNA fragment constructions*

All DNA fragments were amplified by PCR using the Phusion high fidelity polymerase (www.thermoscientificbio.com/) following a protocol as recommended by the manufacturer. Overlapping PCR products were transferred in competence-induced HSISS4 derivatives (Mignolet et al., 2016). *cat*, *erm*, Spec**^R^**, *erm*-*oroP*, P*_xyl1_*, P*_xyl2_*, and *luxAB*-*cat* cassettes were amplified from pNZ5319, pGIUD0855*ery*, pJUD*specmut1-gfp^+^*ter, pSEUDO-P*_usp45_*-*_sf_gfp*(Bs), pZX9, pZX10 and pJIM*cat*, respectively. *comX* and *sptA* mutated promoter were amplified from the WT *comX* and *sptA* *luxAB*-fused promoter strain, respectively. The *sarF-ST* allele was amplified from the pBAD-*sarF-ST*. The full P*_xyl1_-comR*-*spec* at *tRNA^Ser^* locus was amplified in one block from the strain *tRNA^Ser^::*P*_xyl1_-comR*- Spec**^R^** (Mignolet et al., 2018). All the constructed plasmids were sequence-verified.

pBAD-*scuR-ST*. The *scuR*-coding sequence was PCR amplified using the rggD_NcoI SS and rggD_MunI primers. This *scuR* fragment was digested with *Nco*I/*Mun*I and cloned into *Nco*I/*EcoR*I-digested pBAD-*comR-ST* (Mignolet et al., 2018).

pBAD-*sarF-ST*. The *sarF*-coding sequence was PCR amplified using the rggC_NcoI SS and rggC_RI SS primers. This *sarF* fragment was digested with *Nco*I/ *EcoR*I and cloned into *Nco*I/*EcoR*I-digested pBAD-*comR-ST* (Mignolet et al., 2018)

**Supplementary References**

Chopin, A., Chopin, M.C., Moillo-Batt, A., and Langella, P. (1984). Two plasmid-determined restriction and modification systems in *Streptococcus lactis*. Plasmid *11*, 260-263.

Fontaine, L., Dandoy, D., Boutry, C., Delplace, B., de Frahan, M.H., Fremaux, C., Horvath, P., Boyaval, P., and Hols, P. (2010). Development of a versatile procedure based on natural transformation for marker-free targeted genetic modification in *Streptococcus thermophilus*. Appl Environ Microbiol *76*, 7870-7877. AEM.01671-10 [pii]

10.1128/AEM.01671-10

Haustenne, L., Bastin, G., Hols, P., and Fontaine, L. (2015). Modeling of the ComRS Signaling Pathway Reveals the Limiting Factors Controlling Competence in *Streptococcus thermophilus*. Front Microbiol *6*, 1413. 10.3389/fmicb.2015.01413

Lambert, J.M., Bongers, R.S., and Kleerebezem, M. (2007). Cre-lox-based system for multiple gene deletions and selectable-marker removal in *Lactobacillus plantarum*. Appl Environ Microbiol *73*, 1126-1135. AEM.01473-06 [pii]

10.1128/AEM.01473-06

Mignolet, J., Fontaine, L., Kleerebezem, M., and Hols, P. (2016). Complete Genome Sequence of *Streptococcus salivarius* HSISS4, a Human Commensal Bacterium Highly Prevalent in the Digestive Tract. Genome Announc *4*, e01637-01615 4/1/e01637-15 [pii]

10.1128/genomeA.01637-15

Mignolet, J., Fontaine, L., Sass, A., Nannan, C., Mahillon, J., Coenye, T., and Hols, P. (2018). Circuitry Rewiring Directly Couples Competence to Predation in the Gut Dweller *Streptococcus salivarius*. Cell Rep *22*, 1627-1638. S2211-1247(18)30104-9 [pii]

10.1016/j.celrep.2018.01.055

Overkamp, W., Beilharz, K., Detert Oude Weme, R., Solopova, A., Karsens, H., Kovacs, A., Kok, J., Kuipers, O.P., and Veening, J.W. (2013). Benchmarking various green fluorescent protein variants in *Bacillus subtilis*, *Streptococcus pneumoniae*, and *Lactococcus lactis* for live cell imaging. Appl Environ Microbiol *79*, 6481-6490. AEM.02033-13 [pii]

10.1128/AEM.02033-13

Van den Bogert, B., Boekhorst, J., Herrmann, R., Smid, E.J., Zoetendal, E.G., and Kleerebezem, M. (2014). Comparative genomics analysis of *Streptococcus* isolates from the human small intestine reveals their adaptation to a highly dynamic ecosystem. PLoS One *8*, e83418. 10.1371/journal.pone.0083418

PONE-D-13-28051 [pii]
